# Vacuolar Invertase Gene Silencing in Potato (*Solanum tuberosum* L.) Improves Processing Quality by Decreasing the Frequency of Sugar-End Defects

**DOI:** 10.1371/journal.pone.0093381

**Published:** 2014-04-02

**Authors:** Xiaobiao Zhu, Craig Richael, Patrick Chamberlain, James S. Busse, Alvin J. Bussan, Jiming Jiang, Paul C. Bethke

**Affiliations:** 1 Department of Horticulture, University of Wisconsin, Madison, Wisconsin, United States of America; 2 Simplot Plant Sciences, J. R. Simplot Company, Boise, Idaho, United States of America; 3 Vegetable Crops Research Unit, United States Department of Agriculture, Madison, Wisconsin, United States of America; University Paris South, France

## Abstract

Sugar-end defect is a tuber quality disorder and persistent problem for the French fry processing industry that causes unacceptable darkening of one end of French fries. This defect appears when environmental stress during tuber growth increases post-harvest vacuolar acid invertase activity at one end of the tuber. Reducing sugars produced by invertase form dark-colored Maillard reaction products during frying. Acrylamide is another Maillard reaction product formed from reducing sugars and acrylamide consumption has raised health concerns worldwide. Vacuolar invertase gene (*VInv*) expression was suppressed in cultivars Russet Burbank and Ranger Russet using RNA interference to determine if this approach could control sugar-end defect formation. Acid invertase activity and reducing sugar content decreased at both ends of tubers. Sugar-end defects and acrylamide in fried potato strips were strongly reduced in multiple transgenic potato lines. Thus vacuolar invertase silencing can minimize a long-standing French fry quality problem while providing consumers with attractive products that reduce health concerns related to dietary acrylamide.

## Introduction

Potato plants are subjected to a variety of biotic and abiotic stresses that impact plant health, marketable tuber yields and final tuber quality. Poor tuber quality due to the combined effect of environmental and cultural practices in the field can be visualized in finished processed products—the French fry or potato chip [1]–[5]. Suboptimal growing years and cultural practices result in an increase in internal tuber disorders such as brown center, hollow heart, internal necrosis, vascular discoloration and sugar-end defects [6]–[8]. Finished fry products with these disorders must be discarded, constituting an economic loss to the processor. Growers absorb some of the economic burden in the form of contract penalties or rejected raw product. Consumers may be adversely affected as well, in the form of higher prices or decreased product quality.

Sugar-end defect is an internal tuber quality disorder observed primarily in elongate tubers of fry processing potatoes such as Russet Burbank and Ranger Russet [7]. Tubers for fry processing account for 36% of total US potato production [9], and over 50% of the land area for US potato production is planted with these two varieties [10]. Sugar-end defect shows up as a post-fry darkening of one end of the French fry. In the most common type of sugar-end defect, it is tissue from the basal (stem) end of the tuber that fries dark [7]. Darkening is caused by a greater accumulation of reducing sugars at the tuber stem end relative to the tuber apical (bud) end [8], [11], [12]. Reducing sugars react with amino acids during high temperature frying in a non-enzymatic Maillard reaction to form compounds that confer desirable color and flavor to cooked foods [13]. When reducing sugar concentrations are high, as in the case of sugar-end defect tubers, fried products may be unacceptably dark-colored [14] and undesirable to consumers.

Sugar-end defects are typically associated with plants that have had to endure transient periods of high air and soil temperatures during tuber initiation and early tuber bulking [8], [15], [16]. Water deficit at this critical time may also cause or exacerbate sugar-end defect formation [4], [5], [17]. Unlike reducing sugar accumulation during cold-induced sweetening (CIS) [18], reducing sugar accumulation during sugar-end defect formation does not require low temperature storage of tubers. Elevated concentrations of reducing sugars in sugar-end defect tubers cannot be removed by reconditioning, that is by storing at relatively warm temperatures, and this is another difference between sugar-end defects and CIS [7], [19]. Management options used by growers to combat sugar-end defects include ensuring that moisture stress is minimized during early tuber bulking and creating an environment where the canopy is rapidly attained and preserved over the season, which helps to moderate soil temperatures during the day [7]. Postharvest management of sugar-end defect tubers involves extended periods of preconditioning, and early sale where possible [20].

The molecular and biochemical changes that cause sugar-end defects to develop in tubers from stressed plants have not been described completely. Changes in tuber sugar and starch content have been observed during early season heat and water stress, but these differences often do not persist after the stress is removed, and are not observed in tubers at harvest. Increased transcription of *VInv* and a subsequent increase in invertase activity are the immediate causes of sugar-end defects [8]. Vacuolar invertase hydrolyzes the sucrose produced from starch breakdown into one molecule of glucose and one of fructose. These reducing sugars accumulate in tuber cells, and as their concentration increases, defect severity increases. In the most extreme form of sugar-end defect, so-called glassy ends or jelly ends [7], starch content at the tuber stem end is unusually low, giving the tissue a semi-transparent appearance [12], [21]. Reducing sugars accumulate to very high amounts in glassy end tubers, and this makes them unsuitable for processing. Because they lack physical strength, glassy end tubers are prone to damage and often decay from pathogen activity in storage [22].

Three types of invertase isoenzymes are found in plants [23]. Soluble neutral invertases are located in the cytoplasm, whereas cell wall-bound acid invertases and soluble acid invertases play an important role in the apoplastic space and in the vacuole, respectively [23]. Five acid invertase genes, *VInv*/*Pain-1*, *InvGE*, *InvGF*, *InvCD111* and *InvCD141*, were found in potato [24]–[27]. The *VInv*/*Pain-1* gene located on chromosome III encodes a single copy vacuolar invertase. The other four genes are located on chromosome IX or X and encode apoplastic invertases [28]. The cDNA sequence of potato vacuolar invertase was found to be approximately 50% identical with apoplastic invertase cDNA [29]. Both vacuolar and apoplastic invertase isoenzymes have been associated with favorable chip color under a variety of conditions [27], [30]–[32].

Acrylamide is an undesirable by-product of the Maillard reaction that results when reducing sugars react with free asparagine during high temperature cooking [36], [37]. Dietary acrylamide causes cancer and developmental defects in rodents [38]–[40]. In humans, acrylamide is a suspected carcinogen that may have effects on early development [38], [40]–[43]. Reducing sugars are the primary determinants of acrylamide content in fried potato products [40], [44], [45]. Asparagine is the dominant free amino acid in potato tubers and is often present in excess relative to reducing sugars. Several reports have indicated that asparagine, or the ratio of asparagine to total free amino acids, affects the acrylamide content of potato products [46]–[48].

The present study details a method that utilizes the forced down-regulated expression of *VInv* to decrease sugar-end defect formation. This method has been shown previously to control CIS in fry and chip processing potato tubers [33]–[35]. Cold-stored tubers with reduced vacuolar acid invertase activity were shown to have less reducing sugars and produce fried products containing less acrylamide than comparable control products [33]–[35]. Thus, this approach might allow the potato industry to provide consumers with products with improved end product quality that are lower in acrylamide than current products. The utility of *VInv*-silencing to lower the frequency of sugar-end defects has not been shown previously. Data presented here on multiple independently transformed lines of Russet Burbank and Ranger Russet with reduced expression of *VInv* demonstrate the potential for using *VInv*-silencing to reduce sugar-end defect development in fry processing potatoes.

## Materials and Methods

### Development and preliminary screening of *VInv*-silencing lines of Russet Burbank

The previously developed RNAi construct InvBP2 [33] was used to generate a total of 53 RNAi silencing lines of potato cultivar Russet Burbank. This construct uses a CaMV 35S promoter to drive constitutive expression. Leaf tissue was collected from transgenic plants at the four-leaf stage and RNA extracted using the RNeasy Plant Mini Kit (Qiagen) following the manufacturer's instructions. RNA samples were DNAase treated with TURBO DNA-free Kit (Ambion), reverse transcribed with SuperScript III (Invitrogen), and *VInv* expression relative to that of the reference gene *Actin97* was quantified by real-time quantitative PCR using the SYBR Advantage qPCR Premix (Clontech) on MJ Research Opticon 2 (Bio-Rad Laboratories). PCR Primers for the *Actin97* were fp:5′-AGTATGACGAATCTGGTCCTTCTATTG-3′ and rp:5′-ACCCAACAATCAACTCTGCCCTCTC-3′ (amplicon size, 203 bp), and primers for the *VInv* were fp:5′- CATCAAAGACATTTTATGACCCGAA-3′ and rp:5′-TGTGTCCCTGTCTTCTTGTCGTAA-3′ (amplicon size, 154 bp). Twenty-five independent transformants were selected from the 53 original lines for further study based on *VInv* expression in leaves of clones relative to expression in leaves of wild-type Russet Burbank. Multiple clones with a high percentage of *VInv* silencing were selected in order to assess the potential effectiveness of the approach to reduce sugar-end defects. Additional clones with relatively low amounts of *VInv* silencing were included for comparison. Overall, 22 lines showed 80–98% reduction of *VInv* expression and three lines showed 0–80% reduction of *VInv* expression in leaf tissues. Transgenic plants were grown under a 16 h photoperiod using natural light supplemented with sodium vapor lamps as needed to maintain a minimum of 500 μmol m^−2^ s^−1^ photosynthetically active radiation. Tubers were harvested from plants that had senesced naturally and were stored at 4°C for 14 days to stimulate CIS and strong expression of *VInv* in wild-type Russet Burbank. Equal amounts of tissue from positions corresponding to tuber stem end, bud end and middle of the tuber were frozen in liquid nitrogen and ground together. RNA was extracted from tuber tissues using Plant RNA Isolation Mini Kit (Agilent) and quantitative real-time PCR was used to confirm that the *VInv* RNAi-silencing construct was functioning in tubers of the selected lines. Based on relative *VInv* expression in leaves and in cold-stored tubers, five RNAi lines were selected for further analysis because they covered a wide range of *VInv* silencing. These included RBK1, RBK22, RBK25, RBK27, and RBK46 (**Fig. S1**).

### Development of *VInv*-silencing lines of Ranger Russet

The vector, which contains a potato tuber specific ADP glucose pyrophosphorylase promoter, and transformation methods used to create the *VInv*-silenced lines in the Russet Ranger background were described previously [35]. Plant transformations were carried out as described previously [49]. A total of 25 RNAi lines named from 1632-1 to 1632-25 were developed for potato cultivar Ranger Russet. Ten independent lines with low reducing sugar content based on greenhouse grown tubers were assessed under field conditions in 2011 and 2012. Data from the five lines that performed best in 2011, 1632-1, 1632-3, 1632-4, 1632-5, 1632-21, and an empty vector control are presented here.

### Field tuber production and postharvest storage

Tissue culture plantlets of Russet Burbank *VInv*-silencing lines and of Russet Burbank controls were grown in Metromix 360 (Sun Gro Horticulture, Vancouver BC, Canada) in a greenhouse for four weeks and then eight plants of each line were transplanted to research plots at the University of Wisconsin Agricultural Research Station in Hancock, Wisconsin on June 4^th^, 2012. Production practices used were standard for irrigated potato at the Hancock station. Vines were desiccated with two applications of diquat (Reglone, Syngenta) and tubers were harvested by hand on September 14^th^, 2012. For each line, the 20 largest tubers were subsampled with five tubers randomly assigned to each of four sample periods for analysis of gene expression, acid invertase enzyme activity, tuber sugar contents and color uniformity after frying at harvest and after one, three, and five months of cold storage. Harvested tubers were stored in the Hancock Storage Research Facility at 13°C for 47 days for wound healing and preconditioning. The storage temperature was decreased from 13°C to 9°C at a rate of 0.1°C per 8 hours, which took a total of 24 days. Tubers were then held at 9°C, a temperature typical for commercial storage of Russet Burbank.

To produce tubers for molecular and biochemical analyses and seed tubers for field experiments, Ranger Russet transformants were grown for 3 months in Sunshine Mix-1 in 8 liter pots in a greenhouse that was controlled for temperature (18°C minimum and 27°C maximum) and light with a 16 h day and 8 h night photoperiod).

Field trials using untransformed controls, empty vector controls and *VInv*-silenced lines of Ranger Russet were conducted at the University of Idaho Parma Research and Extension Center in Parma, Idaho. Applications of macro and micronutrients followed management recommendations suggested by the University of Idaho. Plots were irrigated using a solid set system with moisture maintained above 65% throughout the growing season. In 2011, each control and transgenic line was represented by 1 plot of 5 hills. In 2012, each control and transgenic line was represented by 5 plots of 20 hills. In both years, in-row spacing was 10 inches with 36 inches between rows. Tubers were harvested 130 to 140 days from planting and stored at 13°C to precondition and encourage wound healing. The final storage temperature of 8°C for tubers of Ranger Russet was achieved by stepping down 0.1°C every 8 hours. Sugar end determinations were made on Ranger Russet tubers stored at 8°C for 1 month. Five randomly selected tubers of lines with the lowest percentages of sugar-end defects in 2012, 1632-1, 1632-3 and 1632-5, and Ranger Russet controls were used for analysis of gene expression and acid invertase enzyme activity using the methods described for Russet Burbank. Tubers (2013 field year) were held at 13°C for 45 days and ramped to 8°C at a rate of 0.33°C per 24 hours. Tubers were held at 8°C for an additional month prior to taking tissue samples for *VInv* quantification and acid invertase activity assays.

### Molecular, biochemical and fried product evaluations for tubers from *VInv*-silencing lines

At the time of harvest and after 1, 3, and 5 months of storage, 1-cm diameter cylinders of tuber tissues were removed from the apical (bud) and basal (stem) ends of five tubers of Russet Burbank and the five Russet Burbank *VInv*-silencing lines. These tissue samples were cut into subsamples of approximately 1 cm length, frozen immediately in liquid nitrogen and used to quantify *VInv* expression, tuber sugar content and acid invertase activity. Gene expression was quantified by qPCR as described above. Acid invertase assays and sugar quantification for Russet Burbank lines used the methods described previously [33]. Sugar quantification of Ranger Russet lines used the method described in Ye et al [35].

Fried strips of Russet Burbank lines were prepared by slicing longitudinal sections 0.95-cm thick and 3-cm wide from the center of individual tubers and cooking in cottonseed oil at 191°C for 3 min 30 s. Acrylamide content of fried strips was assayed as described previously [33]. For Ranger Russet *VInv*-silencing lines grown in 2011, a fry sample consisted of a minimum of twelve pounds of tubers taken from a pooled sample of the 5 hills. In 2012, 20 tubers from a pooled conglomeration from each replicate of 20 hills were used and all 5 replicates were measured. All tubers were cut lengthwise on a 0.95-cm×0.95-cm grid fry knife and the four center strips were fried at 191°C for 3 minutes. Fried strips were laid on a white tray and compared to the USDA Munsell Color Chart for French Fried Potatoes. A sugar-end defect fry had a dark end 0.64 cm long or longer on the darkest two sides of the strip, for the full width of the strip, testing number 3 or darker when compared to the USDA Munsell Color Chart.

### Statistical analysis

For the Russet Burbank *VInv*-silencing lines, relative expression was calculated using Gene Expression Macro software version 1.1 (Bio-Rad Laboratories). All the data for qPCR, tuber sugar contents and acid invertase activity were calculated using the Statistical Analysis System version 9.1 (SAS v9.1) (SAS Institute Inc., Cary, NC, USA). Analyses of variance (ANOVA) were carried out using PROC GLM. Differences in sugar-end defect percentage between the Ranger Russet and transformed lines of Ranger Russet were determined using one-way ANOVA followed by Holm-Sidak multiple comparisons test using GraphPad Prism version 6.0b for Mac (GraphPad Software, La Jolla California).

## Results

### *VInv* expression at the tuber stem and bud ends of Russet Burbank silencing lines

We developed a total of 53 RNAi silencing lines from Russet Burbank. Five silencing lines (RBK1, RBK22, RBK25, RBK27 and RBK46) with a wide range of *VInv* silencing were selected for in-depth analyses (**Fig. S1**). The expression of *VInv* in lines RBK1, RBK22, RBK25 and RBK27 was consistently less than the expression in control Russet Burbank from harvest through five months of storage (**Table 1**). Expression was not consistently reduced in line RBK46, which had lower levels of *VInv* silencing in leaves and cold-stored tubers than the other lines (**Fig. S1**). *VInv* expression in bud end tissue was lowest and reduced 85–96% in line RBK1 at harvest, and one month and three months after harvest. Strongly reduced *VInv* expression was also observed in line RBK22 at harvest and one month and 5 months after harvest. Surprisingly, the constitutive *VInv*-silencing construct was less effective at the tuber stem end than at the tuber bud end (**Table 1**). For each of the five silencing lines, a greater reduction in *VInv* expression relative to Russet Burbank controls was observed at the tuber bud end than at the tuber stem end from the time of harvest to 5 months post harvest, with only one exception. For example, RBK1 had 4% of wild type *VInv* expression in the bud end at harvest, whereas expression in the stem end was 36% wild type (**Table 1**).

**Table 1:** Expression of *VInv* in the bud end and stem end of tubers from *VInv*-silencing lines of Russet Burbank (RBKx) relative to expression of *VInv* in untransformed Russet Burbank at harvest and after one, three and five months of storage.

			Postharvest storage		
Tuber end	Line^§^	Harvest	One month	Three months	Five months
Bud end	Russet Burbank	100%	100%	100%	100%
	RBK46	55%	56%	109%	74%
	RBK1***	4%	8%	15%	25%
	RBK22***	6%	4%	21%	15%
	RBK25**	9%	8%	45%	32%
	RBK27***	11%	5%	13%	20%
Stem end	Russet Burbank	100%	100%	100%	100%
	RBK46	146%	82%	136%	143%
	RBK1**	36%	17%	30%	22%
	RBK22*	84%	37%	36%	23%
	RBK25	68%	30%	50%	38%
	RBK27**	44%	14%	40%	23%

Note: ^§^Asterisks indicate overall differences of least squares means between RBKx and Russet Burbank controls at the same tuber end across sampling times of harvest and one, three and five months of storage.

*, p<0.05;

**, p<0.01;

***, p<0.001.

*VInv* expression was determined using *actin97* as a reference gene and results are presented as a percentage of the level in Russet Burbank controls.

**Figure 1:**
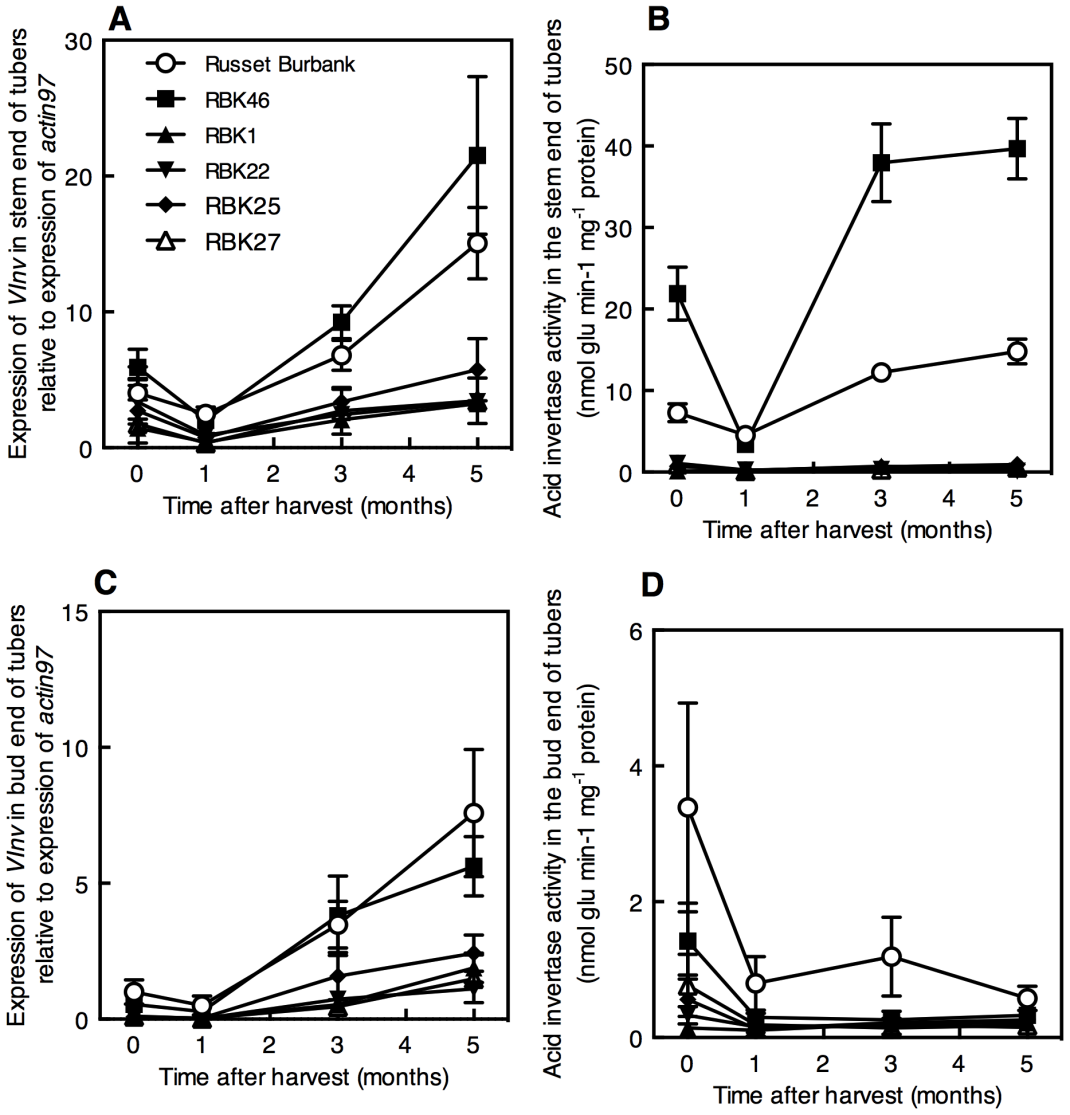
Time and position dependent *VInv* expression and acid invertase activity in Russet Burbank silencing lines differed from that in Russet Burbank controls. *VInv* expression (A, C) and acid invertase activity (B, D) in the stem (A, B) and bud (C, D) end of five *VInv*-silencing lines and controls of Russet Burbank potato tubers at harvest and after 1, 3 and 5 months of storage. *VInv* expression in stem and bud end samples was expressed relative to expression of the reference gene *actin97*. Bars represent mean ± standard error of five independent tuber samples.

*VInv* expression was greater at the tuber stem end than at the bud end for the silencing lines and the Russet Burbank control (**Fig. 1A, C**, p<0.05). *VInv* expression at the tuber stem end of the transgenic lines at harvest was less than in Russet Burbank and RBK46 for lines RBK1, RBK22, RBK25 and RBK27 (**Fig. 1A**). *VInv* expression in the tuber bud end at harvest was less than in Russet Burbank for all of the silencing lines (**Fig. 1C**). *VInv* expression at tuber stem and bud ends decreased during the first month after harvest. The amount of *VInv* transcript in the Russet Burbank control and RBK46 increased dramatically at both ends of the tuber by three and five months of storage, and postharvest expression of *VInv* in RBK46 was not different from that in Russet Burbank controls (**Fig. 1A, C**). *VInv* mRNA abundance in RBK1, RBK22, RBK25 and RBK27 increased much less rapidly with time in storage, and expression was no more than 38% of the Russet Burbank control at both the tuber bud and stem ends five months after harvest.

### Acid invertase activity and reducing sugar accumulation in stored tubers of constitutive Russet Burbank silencing lines

Changes in acid invertase activity at the tuber stem end of Russet Burbank controls and RBK46 (**Fig. 1B**) were consistent with measured changes in *VInv* expression (**Fig. 1A**). Stem end acid invertase activity at harvest and in storage was higher in RBK46 than in the other *VInv*-silencing lines, with the lowest rate of activity observed one month after harvest and higher activity at three and five months after harvest. Russet Burbank controls followed a similar pattern, but activity was less than for RBK46 at harvest and after three and five months of storage. Activity at the tuber stem end of RBK1, RBK22, RBK25 and RBK27 was much less than that of Russet Burbank controls (p<0.0001) and remained low, with activity between 0.11 and 0.93 units mg^−1^ protein at one, three and five months after harvest (**Fig. 1B**).

Acid invertase activity at the tuber bud end was much less than that at the stem end and activity did not change significantly in any of the silencing lines or Russet Burbank controls between one and five months after harvest (**Fig. 1D**, p<0.01). Acid invertase activity in the tuber stem end did not differ from the tuber bud end for lines RBK1, RBK22, RBK25 and RBK27 (p>0.05).

The glucose (**Table 2**), fructose (**Table 3**) and sucrose (**Table 4**) contents of tissues at the stem and bud end of tubers from each of the five Russet Burbank *VInv*-silencing lines were quantified. All of the silencing lines except RBK46 had very low amounts of the reducing sugars glucose and fructose in both ends of the tubers, especially in the stem end, compared with the Russet Burbank control (**Tables 2** **and** **3**). Glucose content at the bud end of RBK1, RBK22, RBk25 and RBK27 was 0.1 mg g^−1^ fresh weight or less from harvest through 5 months of storage, compared to 0.3–1.0 mg g^−1^ fresh weight for Russet Burbank controls (**Table 2**). Glucose at the stem end was consistently 0.2 mg g^−1^ fresh weight or less for lines RBK1, RBK22 and RBK27 while stem end glucose in Russet Burbank was significantly higher at 2.7 to 4.8 mg g^−1^ fresh weight (p<0.01). Tuber fructose content in the silencing lines and Russet Burbank controls was similar to tuber glucose content, although tuber fructose content tended to be less than tuber glucose content (**Tables 2** **and** **3**).

**Table 2:** Glucose content in both ends of Russet Burbank potato tubers and in tubers from *VInv*-silencing lines (RBKx) of Russet Burbank at harvest and after one, three and five months of storage.

Tuber end	Line	Harvest	Postharvest storage		
			One month	Three months	Five months
Bud end	Russet Burbank	1.00±1.08	0.33±0.42	0.36±0.35	0.41±0.72
	RBK46	0.67±0.56	0.34±0.58	0.12±0.13	0.11±0.02
	RBK1	0.06±0.02	0.10±0.12	0.03±0.01	0.05±0.01
	RBK25	0.05±0.03	0.07±0.03	0.08±0.05	0.09±0.04
	RBK27	0.05±0.02	0.06±0.02	0.04±0.01	0.05±0.00
	RBK22	0.07±0.03	0.04±0.01	0.05±0.02	0.06±0.01
Stem end	Russet Burbank	4.80±1.89	4.49±1.95	4.01±3.5	2.66±0.73
	RBK46	4.9±2.41	4.05±2.25	1.98±0.56	3.65±2.30
	RBK1	0.09±0.04	0.06±0.00	0.12±0.10	0.20±0.16
	RBK25	0.44±0.31	0.60±0.54	0.47±0.23	0.66±0.21
	RBK27	0.11±0.07	0.10±0.01	0.14±0.06	0.18±0.08
	RBK22	0.15±0.13	0.10±0.08	0.19±0.09	0.15±0.18

Note: Data are means ± standard deviation of five independent tuber samples. Glucose contents are shown as mg g^−1^ fresh tuber weight.

**Table 3:** Fructose content in both ends of Russet Burbank potato tubers and in tubers from *VInv*-silencing lines (RBKx) of Russet Burbank at harvest and after one, three or five months of storage.

Tuber end	Line	Harvest	Postharvest storage		
			One month	Three months	Five months
Bud end	Russet Burbank	0.67±0.69	0.20±0.31	0.35±0.39	0.36±0.63
	RBK46	0.31±0.38	0.10±0.11	0.10±0.10	0.08±0.016
	RBK1	0.04±0.01	0.03±0.02	0.02±0.01	0.02±0.01
	RBK25	0.04±0.01	0.05±0.02	0.04±0.02	0.04±0.01
	RBK27	0.03±0.02	0.05±0.02	0.03±0.01	0.03±0.00
	RBK22	0.04±0.02	0.03±0.00	0.03±0.00	0.03±0.01
Stem end	Russet Burbank	3.38±1.44	3.66±1.66	2.98±2.23	1.82±0.40
	RBK46	3.53±1.61	3.11±2.39	1.39±0.17	2.40±1.64
	RBK1	0.08±0.03	0.04±0.01	0.07±0.06	0.09±0.05
	RBK25	0.36±0.24	0.53±0.44	0.35±0.22	0.50±0.20
	RBK27	0.07±0.02	0.12±0.02	0.11±0.04	0.12±0.05
	RBK22	0.06±0.03	0.10±0.06	0.10±0.01	0.06±0.02

Note: Data are means ± standard deviation of five independent tuber samples. Fructose contents are shown as mg g^−1^ fresh tuber weight.

**Table 4:** Sucrose content in both ends of Russet Burbank potato tubers and in tubers from *VInv*-silencing lines (RBKx) of Russet Burbank at harvest and after one, three and five months of storage.

Tuber end	Line	Harvest	Postharvest storage		
			One month	Three months	Five months
Bud end	Russet Burbank	2.27±0.39	2.39±0.58	1.42±0.19	1.52±0.19
	RBK46	2.66±0.80	2.24±0.30	1.47±0.15	1.58±0.12
	RBK1	2.90±0.71	2.38±0.48	1.60±0.22	1.58±0.29
	RBK25	1.92±0.51	2.17±0.30	1.55±0.37	1.25±0.18
	RBK27	2.08±0.41	2.30±0.11	1.44±0.17	1.48±0.15
	RBK22	2.91±0.61	2.38±0.19	1.97±0.22	1.78±0.18
Stem end	Russet Burbank	1.42±0.39	1.28±0.13	0.94±0.12	1.13±0.23
	RBK46	1.45±0.30	1.11±0.27	0.97±0.19	1.05±0.23
	RBK1	2.36±0.61	1.60±0.28	1.97±0.61	1.93±0.65
	RBK25	1.50±0.18	1.25±0.19	1.40±0.37	1.50±0.17
	RBK27	2.00±0.43	1.70±0.19	1.96±0.62	2.42±0.24
	RBK22	4.09±2.20	2.04±0.80	3.07±1.03	1.60±0.20

Note: Data are means ± standard deviation of five independent tuber samples. Sucrose contents are shown as mg g^−1^ fresh tuber weight.

Differences in sucrose content were not observed between the tuber bud end and stem end of lines RBK1, RBK22, RBK25 and RBK27 (p>0.05, **Table 4**) tubers in which *VInv* was silenced by more than 50%. However, stem-end sucrose contents in tubers from RBK1, RBK22, and RBK27 were higher than those in RBK25, RBK46, and Russet Burbank control (p<0.05, **Table 4**). At the tuber bud end, however, differences in sucrose content between the silencing lines and the Russet Burbank control were not observed (p>0.05, **Table 4**).

### Constitutive *VInv*-silencing lines of Russet Burbank were free of sugar-end defects

**Figure 2:**
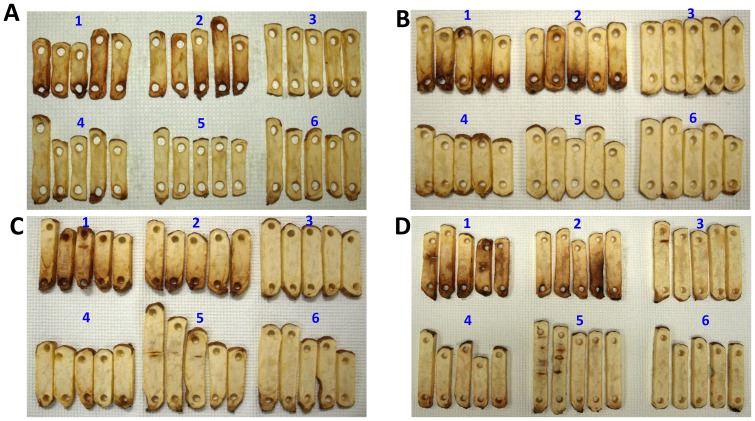
Fried potato slices processed from *VInv*-silencing lines of Russet Burbank potato had fewer sugar end defects than those processed from Russet Burbank controls. Fried strips were prepared from tubers at harvest (A) and after 1 (B), 3 (C) and 5 (D) months of storage. The numbers 1 to 6 represent tubers from Russet Burbank, RBK46, RBK1, RBK25, RBK27, and RBK22, respectively. Slices are positioned with the tuber stem end portion nearest to the bottom of each photograph.

Fried strips of tuber tissue from the *VInv*-silencing lines and Russet Burbank controls were evaluated for dark color in stem-end tissues to estimate the frequency of sugar-end defects. Strips were cut from tubers at harvest and after 1, 3, and 5 months of storage. Russet Burbank controls and RBK46 had dark-color defects at the tuber stem end at each sampling period. Additional dark color was observed around areas where tuber tissue had split and separated during growth or storage (**Fig. 2**). The other four silencing lines had nearly uniform color along the length of the fried strips. For lines RBK1, RBK27 and RBK22, no sugar-end defects were observed at any sampling period, although localized dark areas around tissue that had split were still observed (**Fig. 2**).

### Acrylamide-forming potential of constitutive *VInv*-silencing lines

The acrylamide contents at the tuber stem end of fried strips prepared from Russet Burbank controls and RBK46 were 814 and 762 μg kg^−1^, respectively (**Table 5**). Acrylamide contents in stem end samples from RBK1 and RBK22 were 88 and 122 μg kg^−1^, respectively, and were substantially less than those in control Russet Burbank (p<0.0001). Acrylamide in bud end tissues was also much less in these two silencing lines than in Russet Burbank and RBK46 (p<0.0001). Thus, differences in acrylamide content mirrored observed differences in tuber reducing sugar contents.

**Table 5:** Acrylamide content at stem end and bud end of fried strips prepared from Russet Burbank and three *VInv*-silencing lines three months after harvest.

Line	Tuber end	Acrylamide content (μg kg^−1^)
RBK1	Stem	88±10
RBK22	Stem	122±11
RBK46	Stem	762±41
Russet Burbank	Stem	814±26
RBK1	Bud	58±8
RBK22	Bud	77±6
RBK46	Bud	192±7
Russet Burbank	Bud	387±24

Note: Acrylamide contents are means ± standard deviation of five individual fried strips.

Note that lines RBK1 and RBK22 have strong suppression of *VInv* and RBK46 shows little suppression of *VInv*.

### Tuber-specific *VInv*-silencing reduced sugar-end defects in Ranger Russet lines

**Figure 3:**
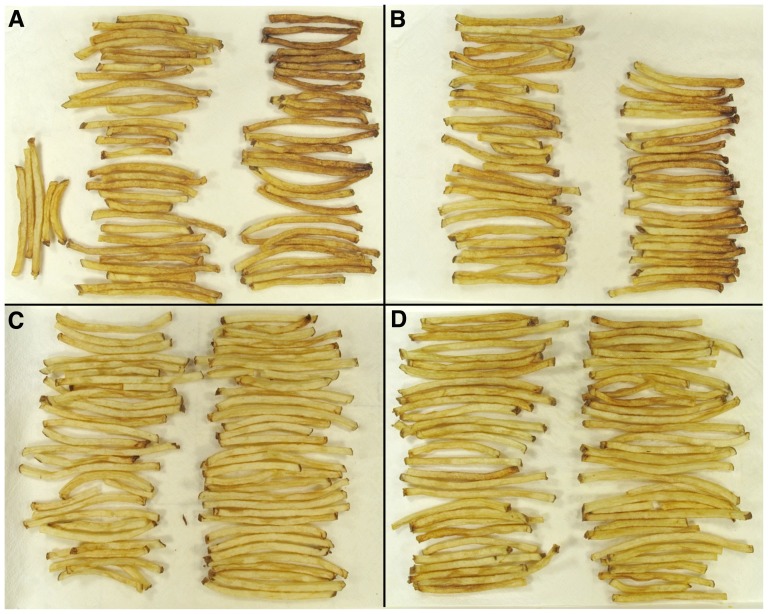
Sugar-end defect frequency was reduced in French fries prepared from *VInv*-silencing lines of Ranger Russet. Sugar-end defects are apparent on nearly half of the fries from Ranger Russet (A) and empty vector (B) control tubers. No sugar-end defect fries where observed in fries from lines 1632-1 (C) and 1632-4 (D) in which the *VInv* had been silenced using RNA interference. In (A) and (B), fries with sugar-end defects are on the right and fries without sugar-end defects are on the left.

**Figure 4:**
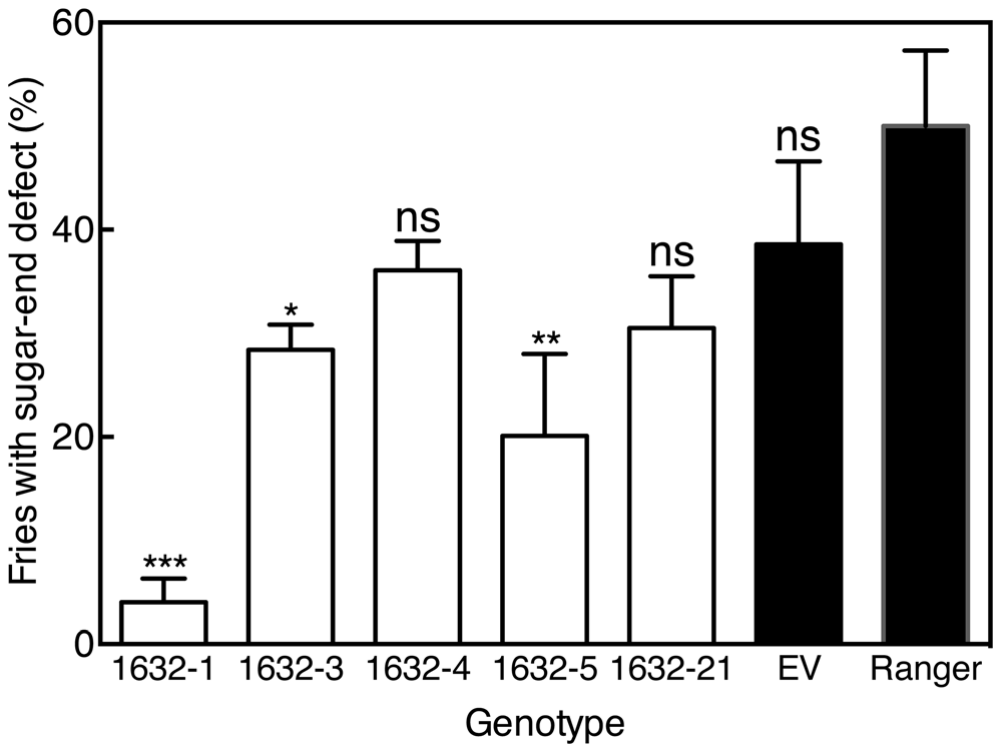
Percentage of French fries with sugar-end defects varied among five lines of Ranger Russet with *VInv*-silencing, empty vector control and Ranger Russet control. Bars represent mean ± standard error of five replicate samples. Differences in means between individual lines and the Ranger Russet control are indicated as not statistically different (ns) or different at the p<0.05 (*), p<0.01 (**), or p<0.001 (***) level.

**Figure 5:**
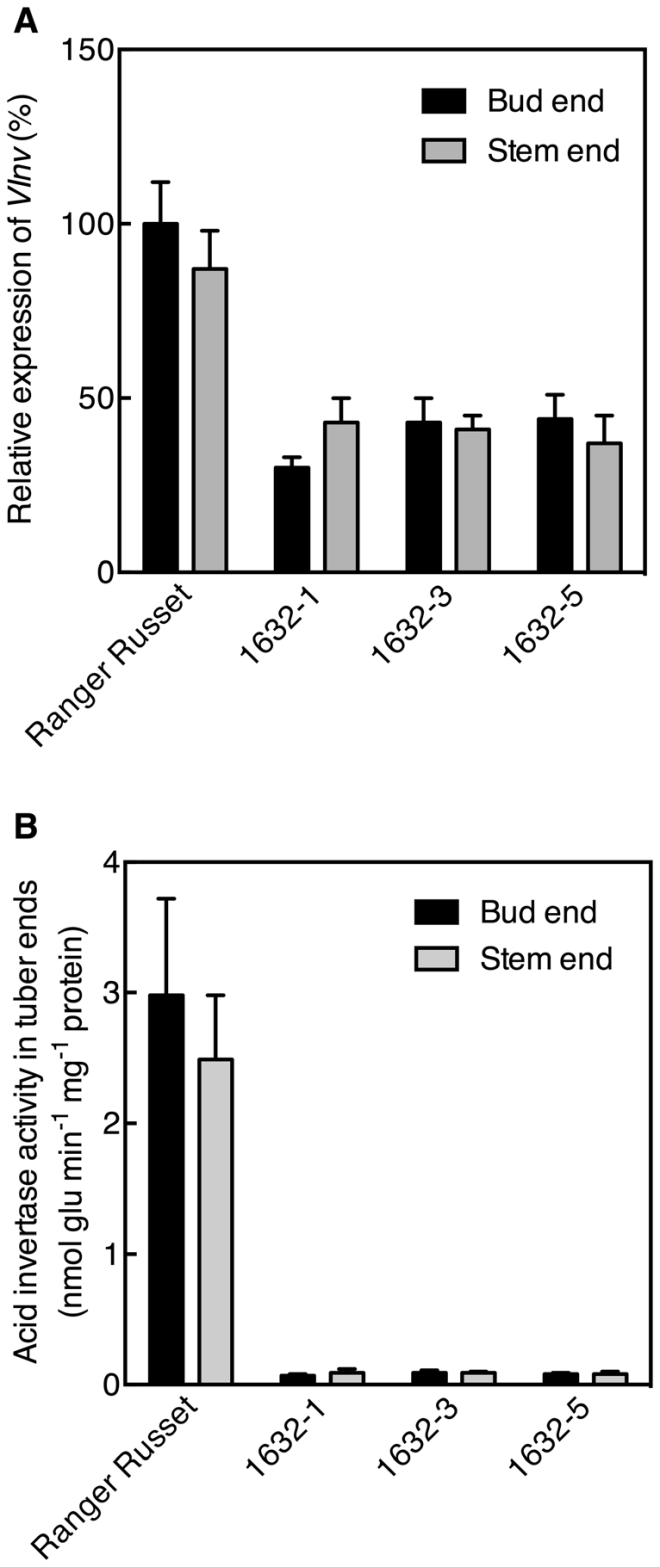
*VInv* mRNA abundance and acid invertase activity were reduced in tubers of Ranger Russet with *VInv*-silencing compared with tubers of Ranger Russet controls. *VInv* expression (A) and acid invertase activity (B) in both tuber ends of RNAi lines 1632-1, 1632-3, and 1632-5 and Ranger Russet control tubers. *VInv* expression relative to expression of the reference gene *actin97* was determined for stem and bud end tuber samples. Expression data are presented as a percentage of *VInv* expression in tuber bud end samples of Ranger Russet controls. Bars represent mean ± standard error of five independent tuber samples.

Constitutive gene silencing using constructs driven by viral promoters such as the CaMV 35S promoter is advantageous in that it is likely to uncover unanticipated effects on plant development and growth that might occur as a result of gene-silencing in tissues other than the target tissue. Constitutive viral promoters are undesirable for commercial applications, however, where target-specific expression using endogenous promoters is preferred. Lines of Ranger Russet in which *VInv* was silenced using the potato tuber specific ADP glucose pyrophosphorylase promoter were evaluated over two field years for susceptibility to sugar-end defect formation. In 2011, a small sample size due to limited seed supply revealed trends toward all silencing lines having reduced sugar-end defects (**Fig. 3**, **Table 6**). Although over 40% of the center strip fries of untransformed control (**Fig. 3A**, Ranger control) and the empty vector control (**Fig. 3B**) showed sugar-end defects, *VInv*-silencing lines all showed dramatically fewer defects ranging from 0% in 1632-1 and 1632-4 to 13.3% in 1632-5 (**Fig. 3C–D** **,** **Table 6**). The same pattern, where the *VInv*-silencing lines showed considerable reductions in sugar-end defects, was observed in 2012 (**Fig. 4**). As in 2011, line 1632-1 had very few defects with an average of 4±2.3% French fries showing sugar-end defects in 2012. Other lines showed less of a reduction in 2012 than in 2011, but the percentage of sugar-end defect fries was less in lines 1632-3 and 1632-5 than in the Ranger Russet control (**Fig. 4**). Gene silencing strongly reduced *VInv* expression and acid invertase activity in both ends of silencing lines 1632-1, 1632-3, and 1632-5 compared with control Ranger Russet tubers (p<0.01, **Fig. 5**). *VInv* expression in these three lines was 30–50% of that in Range Russet controls and acid invertase activity was 2–4% of that in Ranger Russet controls (**Fig. 5**).

**Table 6:** The frequency of center cut French fries with sugar-end defects (SE) from *VInv*-silencing lines of Russet Ranger (1632-x), empty vector control and untransformed (Ranger control) Ranger Russet tubers.

Line ID	Number of fries scored	Number of fries with SE	Percent fries with SE
1632-1	60	0	0
1632-3	60	4	6.7
1632-4	60	0	0
1632-5	60	8	13.3
1632-21	60	4	6.7
Empty vector	51	24	47
Ranger control	60	25	42

## Discussion

The data presented here demonstrate that *VInv*-silencing is an effective method for reducing the frequency of sugar-end defects in processing potatoes. Experiments were done with multiple transgenic lines of the two most widely grown fry processing cultivars in North America. Field grown tubers for analysis were generated from seed tubers and from tissue culture plantlets. Lines in which *VInv* was strongly silenced relative to untransformed controls consistently had lower concentration of reducing sugars at the basal portion of the tuber and fewer sugar-end defects than controls.

Silencing *VInv* effectively controlled sugar-end defects, but was not sufficient to prevent all post-harvest reducing sugar accumulation (**Tables 2** **,** **3**). This is consistent with previous data obtained using chip and fry processing potatoes stored at cold temperatures [23]–[25]. In those cases, responses associated with CIS including greater accumulation of vacuolar invertase messenger RNA, increased acid invertase activity and greater tuber reducing sugar concentrations, were inhibited most strongly in lines with greatest *VInv* silencing [33]–[35]. Likewise, responses to in-season environmental stress that resulted in sugar-end defect formation were reduced by *VInv* silencing. Tubers from some lines of Ranger Russet exhibited higher rates of sugar-end defects in 2012 than in 2011, and this is likely attributable to differences in the growing season. Severity of sugar-end defects is known to fluctuate from year to year in a given location depending on the environmental conditions, especially temperature during the early tuber-bulking period.

*VInv* silencing at the tuber stem end was less effective than at the tuber bud end in each of the Russet Burbank transgenic lines (**Table 1**). Suppression of *VInv* expression relative to controls was highly effective at the tuber bud end, up to 96% suppression, but was much less effective in stem end tissues of the same tubers. For example, RBK1 at harvest had 4% of control *VInv* mRNA at the tuber bud end and 36% of control *VInv* mRNA at the tuber stem end. Multiple causes for this can be suggested, including apical (bud) to basal (stem) differences in post-transcriptional mRNA stability and context-dependent efficiency of the silencing construct. Tuber bud end and stem end tissues also differed in the correspondence, or lack thereof, between changes in *VInv* mRNA abundance and changes in acid invertase activity. In stem end tissues of control Russet Burbank and line RBK46, changes in *VInv* mRNA accumulation through 5 months of storage were mirrored by changes in enzyme activity. This was not observed in bud end tissues of Russet Burbank and RBK46 tubers. Pronounced increases in *VInv* mRNA occurred between 1 and 5 months of storage, but acid invertase activity did not change in either of those lines during that period. It may be that relatively small increases in *VInv* mRNA, i.e. expression relative to *actin97* of less than approximately 5, do not result in an increase in acid invertase activity at either end of the tuber. This suggestion is supported by the data from RBK1, RBK22, RBK25 and RBK27 at the tuber stem end and from all *VInv*-silencing lines and the Russet Burbank control at the tuber bud end.

Differences in tuber bud and stem end sucrose content are often observed in Russet Burbank and this difference has been attributed to a higher rate of vacuolar invertase activity at the tuber stem end compared with the tuber bud end [7], [8]. The observation that differences in sucrose content were not observed between the tuber ends in silencing lines of Russet Burbank in which *VInv* expression was decreased by more than 50% is consistent with this idea.

The dark color observed after frying was associated with a substantial increase in overall acrylamide content of the fried strips (**Table 5**). Acrylamide content was 427 and 570 μg kg^−1^ greater on the stem end than on the bud end of fried strips for Russet Burbank controls and RBK46, respectively. Since each strip was cut in half prior to acrylamide analysis, average acrylamide content for intact strips would be approximately the mean of bud and stem end samples. The mean value for Russet Burbank was approximately 600 μg kg^−1^, which equals the indicative value for acrylamide in Europe as established by the European Commission [50]. Mean acrylamide contents of fried strips for RBK1 and RBK 22 were just 12% and 17%, respectively, of that for Russet Burbank. Decreased acrylamide content in these two lines was likely related to similar decreases in *VInv* mRNA, acid invertase activity, and reducing sugar content in both tuber ends compared to those of Russet Burbank and RBK46.

Silencing of the asparagine synthetase-1 gene provides an alternative approach to reduce the acrylamide content in processed potato products [51], but this approach does not address the CIS and sugar-end defects problems. One way to reduce the acrylamide content of fried potato products to as low as reasonably achievable may be to use transgenic lines in which both the *VInv* and asparagine synthetase-1 genes are silenced.

Ranger Russet *VInv*-silencing lines demonstrated that tuber specific gene silencing can be used to decrease sugar-end defect formation. For all of the Ranger Russet lines described, tubers harvested in 2011 and 2012 and stored at 8°C produced fries with acceptable color in tissue corresponding to the tuber bud end. However, a very stressful growing season in 2012 demonstrated a requirement for very effective silencing if sugar-end defects are to be managed routinely. Of the lines described, only 1632-1 had very low incidence of sugar-end defects in 2012, although 1632-3 and 1632-5 had fewer sugar-end defects than Ranger Russet controls.

Dark fry color was not directly addressed in this research on sugar ends. A number of stresses that occur in the field can cause an increase in reducing sugars throughout the tuber. Tuber sugar contents are influenced by many factors including disease stress, temperature and water stress, management practices and mechanical handling. In cases where such stresses occur, the consequence is usually an increase in fry color distributed over the length of the French fry strip. Defects such as these are also likely to be reduced by silencing of *VInv*, but further evaluation of the *VInv*-silenced lines described in this research is needed in order to show this conclusively.

In summary, the data presented here show that silencing the *VInv* can be used to reduce the incidence of sugar-end defects in fry processing potatoes. Success of this approach depends on the extent of *VInv*-silencing, with more effective silencing at the tuber stem end producing fewer defects. Decreasing sugar-end defect frequency could benefit potato growers and processors by reducing rejections at the processing plant, and providing a more reliable supply of raw product. Consumers also benefit, in that low vacuolar invertase lines produce attractive end products that are potentially much lower in acrylamide than comparable products made from control varieties.

## Supporting Information

Figure S1 Expression of *VInv* in potato leaf and tuber tissues varied between Russet Burbank *VInv*-silencing lines and Russet Burbank controls. *VInv* expression was determined using *actin97* as a reference gene and presented as a percentage of the level in Russet Burbank controls. Potato leaf tissues were collected from transgenic plants at the four-leaf stage and tuber tissues were collected from cold-stored tubers (14 d of storage at 4°C). Data are presented for five lines with different degrees of *VInv* silencing. Bars represent mean ± standard error of two independent leaf samples and three independent tuber samples.
(TIF)
