# Supplementary figures and images for: Vacuolar Invertase Gene Silencing in Potato (Solanum tuberosum L.) Improves Processing Quality by Decreasing the Frequency of Sugar-End Defects
Source: PLoS One. 2014 Apr 2;9(4):e93381. doi: 10.1371/journal.pone.0093381 (PMC3973568; doi:10.1371/journal.pone.0093381)

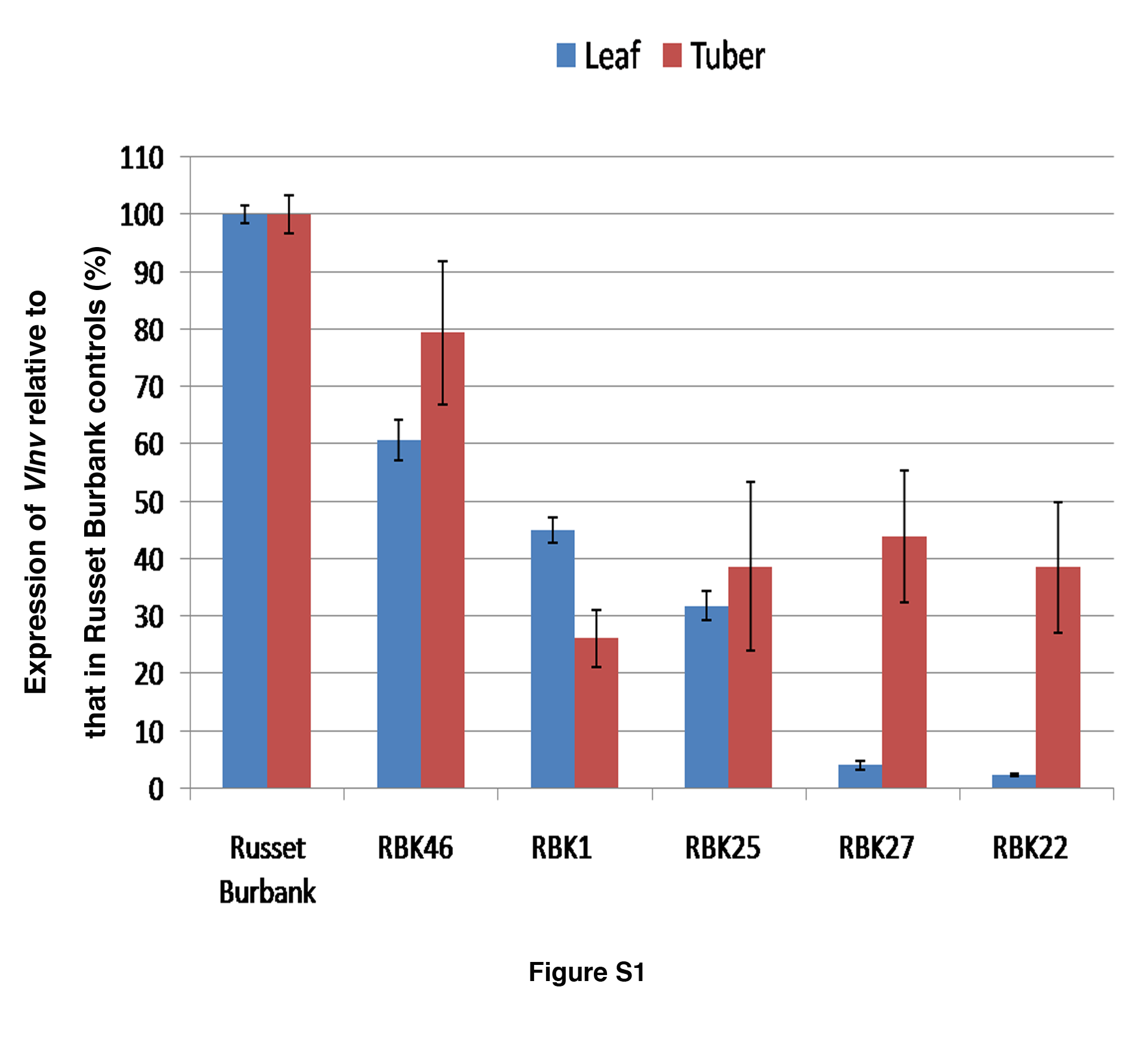

Supplement: Figure S1 — Expression of VInv in potato leaf and tuber tissues varied between Russet Burbank VInv-silencing lines and Russet Burbank controls. VInv expression was determined using actin97 as a reference gene and presented as a percentage of the level in Russet Burbank controls. Potato leaf tissues were collected from transgenic plants at the four-leaf stage and tuber tissues were collected from cold-stored tubers (14 d of storage at 4°C). Data are presented for five lines with different degrees of VInv silencing. Bars represent mean ± standard error of two independent leaf samples and three independent tuber samples. (TIF) [file pone.0093381.s001.tif]
